# Using Medical Loss Ratio Data to Examine Advance Premium Tax Credits

**DOI:** 10.1001/jamahealthforum.2025.5896

**Published:** 2025-12-26

**Authors:** Elizabeth Plummer, Joshua Brooker, Mark Meiselbach, Ge Bai

**Affiliations:** 1 Texas Christian University Neeley School of Business, Fort Worth; 2 Texas Christian University Burnett School of Medicine, Fort Worth; 3 Individual Markets Working Group, National Association of Benefits and Insurance Professionals, Lancaster, Pennsylvania; 4 Johns Hopkins Bloomberg School of Public Health, Baltimore, Maryland; 5 Johns Hopkins Carey Business School, Baltimore, MD; 6 Johns Hopkins Bloomberg School of Public Health, Baltimore, MD

## Abstract

This economic evaluation examines trends in enrollment, premiums, and advance premium tax credits for insurance plans.

## Introduction

In 2025, more than 24 million people were enrolled in individual marketplace health plans.^1^ The advance premium tax credit (APTC), established by the Patient Protection and Affordable Care Act (ACA), subsidizes low-income households that purchase qualified individual marketplace health plans by transferring funds directly to insurers. From 2021 through 2025, Congress temporarily expanded APTC eligibility to households with incomes greater than 400% of the federal poverty level.

Prior analyses of APTCs were based on Congressional Budget Office (CBO) projections or Internal Revenue Service data, which present challenges, including multiyear delays in data availability, enrollee reporting errors, and reliance on point in time or average enrollment numbers.^2,3^ To quantify APTC amounts and examine their association with insurance premiums, we used medical loss ratio (MLR) reports from health insurers, required annually from all insurers participating in ACA marketplaces. We analyzed 10-year trends (2014-2023) in enrollment, premiums, and APTC amounts for insurance plans by whether plans received APTCs.

## Methods

ACA requires private health insurers to submit annual reports to the US Department of Health and Human Services for each state where they sell ACA-compliant individual plans through federal or state exchanges. Our sample included all ACA-compliant individual plan-years reporting positive premiums and enrollment (measured as life-years, including dependents) for 2014 (first year of APTCs) through 2023 (most recent year available).^4^

For each year, we examined enrollment, premiums, and APTC amounts separately for plans that received APTCs (APTC plans) vs those that did not (non-APTC plans). All dollar amounts were adjusted for inflation and expressed in 2023 dollars (eMethods 1 and 2 in Supplement 1).

This study followed the Strengthening the Reporting of Observational Studies in Epidemiology (STROBE) reporting guidelines. No institutional review board approval was sought because no human participants were involved. The analysis was conducted using SAS, version 9.4 (SAS Institute).

## Results

**Figure 1.**
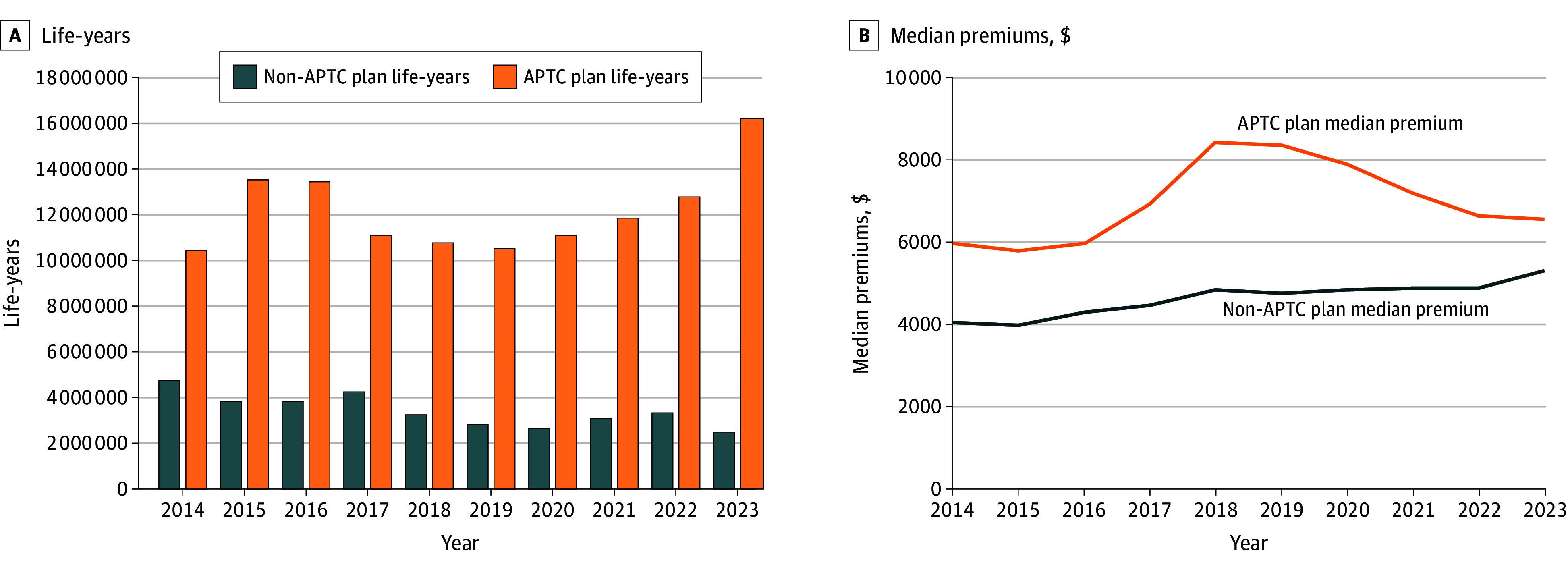
Total Number of Life-Years and Median Premiums for Advance Premium Tax Credit (APTC) and Non-APTC Plans From 2014 to 2023^a,b^ ^a^All dollar amounts were adjusted to 2023 values using the Consumer Price Index. ^b^Median premium is the median value of premium per life-year, which is calculated using all APTC (non-APTC) plans in a given year. All dollar amounts were adjusted to 2023 values using the Consumer Price Index.

Annual enrollment for non-APTC plans declined 47% from 4.7 million in 2014 to 2.5 million in 2023 (Figure 1). Annual enrollment in APTC plans fluctuated but increased 54% from 10.5 million in 2014 to 16.2 million in 2023. Nearly 90% of individual plan enrollment in 2023 was in APTC plans.

From 2014 to 2023, the median premium per life-year for non-APTC plans increased 30% ($4064 to $5306). For APTC plans, it fluctuated, peaking in 2018 ($8422), and was higher than that for non-APTC plans during every year.

**Figure 2.**
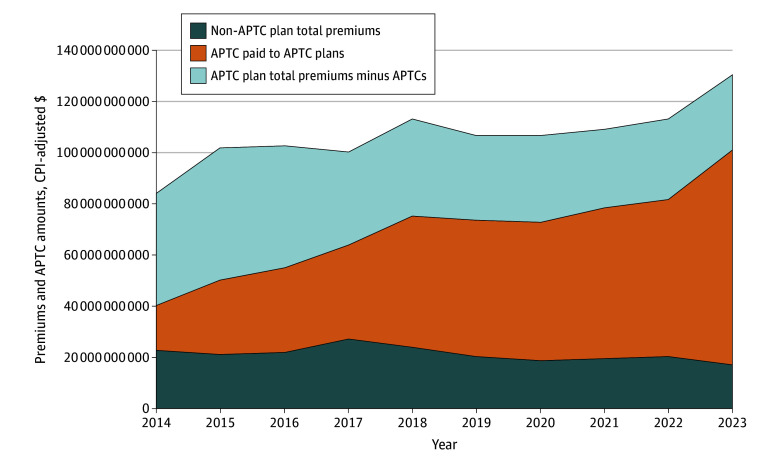
Total Premiums and Advance Premium Tax Credits (APTCs) for APTC and Non-APTC Plans From 2014 to 2023^a^ ^a^All dollar amounts were adjusted to 2023 values using the Consumer Price Index (CPI).

Total premiums summed across all non-APTC plans decreased 23% from $22.6 billion in 2014 to $17.4 billion in 2023 (Figure 2). In contrast, total premiums for APTC plans increased 83% from $61.8 billion to $113 billion, with APTCs playing an increasingly important role in funding premiums. In 2014, APTCs accounted for 28.6% of total premiums for APTC plans. During the next 9 years, APTC payments grew at a compound annual rate of 18.8% (370% cumulative increase), reaching nearly 74% of total APTC plan premiums in 2023. Annual APTCs and premiums were highly correlated (Pearson correlation, 0.946).

## Discussion

We used mandatory MLR filings that reported insurers’ APTC receipts to quantify enrollment, premiums, and APTC amounts for APTC plans and non-APTC plans from 2014 to 2023. APTCs totaled $61.4 billion and $83.2 billion in 2022 and 2023, respectively, compared with CBO reports showing premium tax credits totaling $60 billion and $71 billion, respectively.^5^ This suggests that the 2023 estimate from the CBO was understated by 17%.

Spikes in APTC plan enrollment and payments since 2022 are associated with policy changes that expanded APTC eligibility. If the enhanced APTC subsidies expire, as scheduled in December 2025, some current enrollees, facing higher premiums, will likely opt out of exchange plans, destabilizing the individual market. Estimating and quantifying the effects of expiring APTC subsidies is beyond this study’s scope and remains a topic for future research.

MLR filings may understate or overstate APTC amounts if enrollees claim additional premium tax credits or owe repayments when filing tax returns. These misstatements have accounted for no more than 3% of total APTC amounts.^6^

## Notes

Supplement 1. eMethods 1. Defining APTC and non-APTC plans—measurement error eMethods 2. Insurers in merged markets

Supplement 2. Data sharing statement
