# Supplementary material for: Using Medical Loss Ratio Data to Examine Advance Premium Tax Credits
Source: JAMA Health Forum. 2025 Dec 26;6(12):e255896. doi: 10.1001/jamahealthforum.2025.5896 (PMC12743276; doi:10.1001/jamahealthforum.2025.5896)
Supplement: Supplement 1. — eMethods 1. Defining APTC and non-APTC plans—measurement error eMethods 2. Insurers in merged markets [file jamahealthforum-e255896-s001.pdf]

Supplemental Online Content

Plummer E, Brooker J, Meiselbach M, Bai G. Use of medical loss ratio data to examine advanced premium tax credits from 2014 to 2023. *JAMA Health Forum*. 2025;6(12):e255896. doi:10.1001/jamahealthforum.2025.5896

eMethods 1. Defining APTC and non-APTC plans—measurement error

eMethods 2. Insurers in merged markets

This supplemental material has been provided by the authors to give readers additional information about their work.

© 2025 Plummer E et al. *JAMA Health Forum*.

eMethods 1. Defining APTC and non-APTC plans – Measurement Error

The MLR filings include issuers that provide any health insurance coverage subject to the MLR requirement (i.e., both on-exchange and off-exchange plans). Non-ACA compliant plans are not included in the MLR reports.

If an insurer (i.e., HIOS Issuer ID) offers both on-exchange and off-exchange plans, all data will be combined into a single column for MLR filing and reporting purposes. If the insurer reports a positive APTC amount received, we code this as an “APTC plan.” If no APTC amount is reported, we code this as a “non-APTC plan.”

This can introduce measurement error if enrollees are incorrectly categorized into the wrong plan type. Specifically:

1. If an insurer offers both on-exchange and off-exchange plans, and also reports receiving APTCs, these will be categorized as “APTC plans.” Accordingly, our categorization will *overstate* the estimated number of persons enrolled in APTC plans and *understate* the number of persons enrolled in non-APTC plans (Figure 1). Our categorization will also *overstate* the total premiums paid by APTC plans (Figure 2), meaning the APTC subsidy is a larger percentage of APTC-plan premiums than is shown in Figure 2.

2. Alternatively, if an insurer offers on-exchange plans (and possibly off-exchange plans) but reports no APTCs because it does not have members receiving PTCs due to income ineligibility, these will be categorized as “non-APTC plans.” Accordingly, our categorization will *overstate* the estimated number of persons enrolled in non-APTC plans and *understate* the number of persons enrolled in APTC plans (Figure 1). This will also *understate* the total premiums paid by APTC plans (Figure 2), meaning the APTC subsidy is a smaller percentage of APTC-plan premiums than is shown in Figure 2.

© 2025 Plummer E et al. *JAMA Health Forum*.

eMethods 2: Insurers in Merged Markets

We obtained the data used in our study from Part 1 and Part 2 of the MLR filings, which report individual and small group market information separately. Issuers of health insurance coverage in the individual and small group markets that merge their markets in accordance with state law (such as Massachusetts, Vermont, and D.C.) only combine markets for purposes of Part 3 of the MLR filing (i.e., the MLR and rebate calculation).

© 2025 Plummer E et al. *JAMA Health Forum*.
