# Supplementary material for: Using Medical Loss Ratio Data to Examine Advance Premium Tax Credits
Source: JAMA Health Forum. 2025 Dec 26;6(12):e255896. doi: 10.1001/jamahealthforum.2025.5896 (PMC12743276; doi:10.1001/jamahealthforum.2025.5896)
Supplement: Supplement 2. — Data sharing statement [file jamahealthforum-e255896-s002.pdf]

# Data Sharing Statement

Plummer. Using Medical Loss Ratio Data to Examine Advanced Premium Tax Credits. *JAMA Health Forum*. Published December 26, 2025. doi:10.1001/jamahealthforum.2025.5896

**Data**
**Data available:** No

**Additional Information**
**Explanation for why data not available:** The data used in this study is publicly available.
